# Addressing the Concern of Orange-Yellow Fungus Growth on Palm Kernel Cake: Safeguarding Dairy Cattle Diets for Mycotoxin-Producing Fungi

**DOI:** 10.3390/microorganisms12050937

**Published:** 2024-05-05

**Authors:** Carlos Bastidas-Caldes, David Vasco-Julio, Maria Huilca-Ibarra, Salomé Guerrero-Freire, Yanua Ledesma-Bravo, Jacobus H. de Waard

**Affiliations:** 1 One Health Research Group, Facultad de Ingeniería y Ciencias Aplicadas, Biotecnología, Universidad de Las Américas, Quito 170530, Ecuador; carlos.bastidas@udla.edu.ec (C.B.-C.); paula.huilca@outlook.com (M.H.-I.); or monica.guerrero@udla.edu.ec (S.G.-F.); yanua.ledesma@udla.edu.ec (Y.L.-B.); 2 Posgrado en Ciencias Biológicas, Unidad de Posgrado, Edificio D, 1° Piso, Circuito de Posgrados, Ciudad Universitaria, Ciudad de México 04510, Mexico; david.vasco.julio@outlook.com; 3 Centro de Investigación Sobre Enfermedades Infecciosas, Instituto Nacional de Salud Pública, Cuernavaca 62050, Mexico; 4 Programa de Doctorado, Facultad de Ciencias Veterinarias, Universidad de Buenos Aires, Buenos Aires C1063ACV, Argentina

**Keywords:** mycotoxins, yeast, palm kernel cake, surveillance, *Geotrichum candidum*, *Pichia kudriavzevii*, *Candida ethanolica*

## Abstract

Palm kernel cake (PKC), a byproduct of palm oil extraction, serves an important role in Ecuador’s animal feed industry. The emergence of yellow-orange fungal growth in PKC on some cattle farms in Ecuador sparked concerns within the cattle industry regarding a potential mycotoxin-producing fungus on this substrate. Due to the limited availability of analytical chemistry techniques in Ecuador for mycotoxin detection, we chose to isolate and identify the fungus to determine its association with mycotoxin-producing genera. Through molecular identification via ITS region sequencing, we identified the yellow-orange fungus as the yeast *Candida ethanolica*. Furthermore, we isolated two other fungi—the yeast *Pichia kudriavzevii*, and the fungus *Geotrichum candidum.* Molecular identification confirmed that all three species are not classified as mycotoxin-producing fungi but in contrast, the literature indicates that all three have demonstrated antifungal activity against *Aspergillus* and *Penicillium* species, genera associated with mycotoxin production. This suggests their potential use in biocontrol to counter the colonization of harmful fungi. We discuss preventive measures against the fungal invasion of PKC and emphasize the importance of promptly identifying fungi on this substrate. Rapid recognition of mycotoxin-producing and pathogenic genera holds the promise of mitigating cattle intoxication and the dissemination of mycotoxins throughout the food chain.

## 1. Introduction

Over the past few decades, Ecuador has witnessed the remarkable growth of its cattle and dairy industry, with a substantial increase to nearly four and a half million animals [1]. Simultaneously, approximately 225,000 hectares of the country’s tropical regions have been dedicated to oil palm cultivation. This extensive monoculture venture constitutes a noteworthy 4% of Ecuador’s agricultural production, yielding an annual output of around 2,418,855 tons of palm oil [1]. Within the ambit of this booming industry, an essential byproduct produced from the palm oil extraction process is palm kernel cake (PKC). This residual substance, left behind after the extraction of oil from palm kernels, is considered a valuable source of protein and fiber. Its significance is emphasized by its widespread utilization as an ingredient in animal food, serving the nutritional needs of dairy cattle and various other livestock [2,3,4]. 

Mycotoxins are toxic compounds with a low molecular weight that are produced as secondary metabolites by specific filamentous fungi. These mycotoxin-producing fungi are widely recognized in the agricultural industry and can contaminate staple products like corn, peanuts, and rice [5,6]. Principally, fungi belonging to the *genera Aspergillus*, *Penicillium*, *Fusarium*, and *Alternaria* are known for their ability to produce significant mycotoxins [7]. Among these mycotoxins, aflatoxins stand out due to their potency as some of the most potent natural toxins, associated with carcinogenic and teratogenic properties [7,8].

Examples of mycotoxin contamination have been observed in food crops and stored food within tropical and subtropical climates, including mycotoxin presence in peanuts from Malaysia, India, and Nigeria, as well as in corn from Saudi Arabia and South Africa [9,10]. PKC, the residual byproduct from the palm oil industry, is no exception, and it has been established that this cattle food supplement can serve as a substrate for the growth of mycotoxin-producing fungi [11,12,13], causing growing concern within the dairy industry. Mycotoxin contamination in cattle feed can have a significant risk to both calves and dairy cattle, leading to acute and chronic disorders in animal production and substantial economic losses. In addition to this direct effect, there is a risk of transmission of mycotoxins to humans through products derived from these cattle, such as milk and cheese [7,14,15]. 

**Figure 1:**
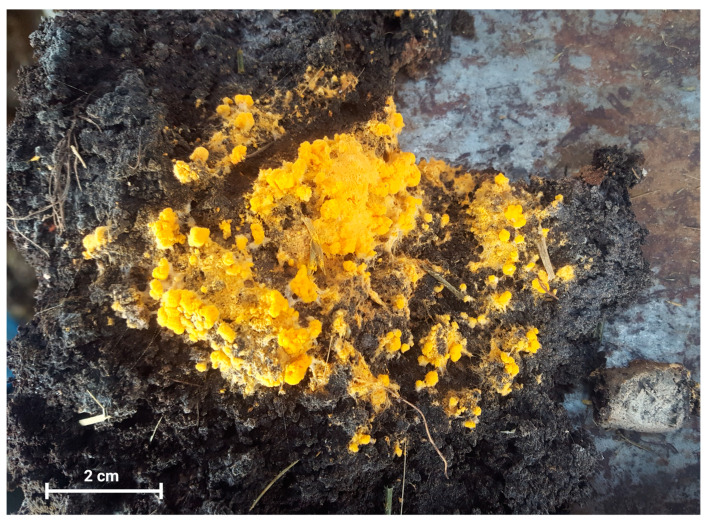
Growth of a yellow-orange fungus on the palm kernel cake used to feed cattle on a farm in a tropical region of Ecuador.

Notably, between 2020 and 2021, there was a significant rise in calf mortality in tropical areas of Ecuador where PKC is commonly used as animal feed, prompting an industry-wide alert regarding mycotoxin presence. However, the root cause of these deaths was never thoroughly investigated. Also, in the same year, cattle ranchers in Ecuador raised concerns about a yellow-orange fungal intrusion in PKC storage facilities (as shown in Figure 1), suggesting that this growth indicates the presence of pathogenic or mycotoxin-producing fungi potentially harmful to their cattle. As a result, they requested a thorough investigation into the matter.

Several techniques are regarded as the gold standard for mycotoxin detection in food, including high-performance liquid chromatography (HPLC), or liquid chromatography–electrospray ionization–tandem mass spectrometry (LC-ESI-MS/MS) [16,17]. These methods can be employed in various food sources such as corn, rice, and other seeds. However, they can be costly and/or inaccessible in rural areas, posing a significant limitation, especially for low-income countries. Moreover, the chemical diversity and co-occurrence of mycotoxins, their different concentrations in agricultural products and complex food matrices with mycotoxin contamination require special extraction, clean-up, and detection methods. No laboratory in Ecuador has yet standardized this for the sample in question. 

Alternative strategies for strengthening the surveillance of mycotoxin-producing fungi have been developed using molecular biology techniques, such as a multiplex polymerase chain reaction (PCR) that can detect the four major mycotoxin metabolic pathway genes, nor1 (aflatoxin), Tri6 (trichothecene), FUM13 (fumonisin), and otanps (ochratoxin), using culture isolates [18]. Another alternative approach involves the direct detection of mycotoxin-producing fungal genera or species by identifying genera or species-specific genes using PCR. [19,20]. Due to the limited availability of HPLC or LC-ESI-MS/MS techniques in Ecuador, we opted for the latter option and decided to identify the predominant fungus, distinguished by its striking coloration, through molecular techniques involving PCR and sequencing. We extracted DNA from primary cultures and the yellow-orange spores produced by this fungus to facilitate their respective identification. The aim of our research was to determine whether the fungus with the alarming orange-yellow spores was non-harmful and unrelated to a genus known for producing mycotoxins.

## 2. Materials and Methods

### 2.1. Area of Study and Sampling

Farms located in the tropical regions of Ecuador, specifically in Santo Domingo de los Tsáchilas and Los Ríos provinces, were chosen for sampling. These farms play a pivotal role in a significant dairy industry that relies on palm kernel cake (PKC) for calf feeding. A PKC storage outbuilding exhibiting conspicuous yellow-orange fungal growth was singled out as the sampling site. 

Using a small gardening shovel, we collected a portion of the whitish mycelium, along with the palm cake substrate, and placed them in sterile 15 mL Falcon tubes. To gather the orange-yellow spores, we gently agitated the shovel near the Falcon tubes until approximately 100 µL of orange particles was deposited inside. Subsequently, all samples were transported at room temperature to the research laboratories at Universidad De Las Americas in Quito, Ecuador, for further examination. 

### 2.2. Fungi Culture

In the laboratory, the mycelium was cultured by placing small fragments of approximately 2 mm^2^ of the visible mycelium structure onto Sabouraud Dextrose Agar (SDA) medium using a sterile inoculation loop. The fragments were gently pressed onto the surface of the plate. To enhance selectivity against commensal microorganisms, chloramphenicol (50 mg/L), nalidixic acid (25 mg/L), and vancomycin (10 mg/L) were added to the culture medium.

Spores were cultured by inoculating the yellow-orange spores, stored in the 15 mL Falcon tube, onto SDA medium with antibiotics using a sterile swab. The samples were then incubated at 25 °C for a period of 15 days. To determine whether the isolated fungi could utilize PKC for growth, a re-isolation test was performed in which fungi previously cultured on SDA were re-isolated on sterile PKC under the same culture conditions previously used for the isolation on SDA.

### 2.3. Molecular Method for the Identification of the Fungi

DNA was extracted from the sampled spores and cultured mycelium using the following procedure: a sterile bacteriological loop inoculated spores or cultured mycelium into a 1.5 mL Eppendorf tube containing 200 µL of TE solution (10 mM Tris-HCl and 1 mM EDTA, pH 8.0). The tube was then incubated at −20 °C overnight. Following incubation, the samples underwent sonication in an Ultrasonic Water Bath 5.7 L CPX3800 Fisherbrand^TM^ (Fisher Scientific, Waltham, MA, USA) for 30 min. After sonication, the tubes were placed on ice for 10 min, heated at 95 °C for 5 min, refrigerated at −20 °C for 15 min, and vortexed three times. Subsequently, they were centrifuged at approximately 1450 rad/s (14,000 rpm) for 1 min, and the supernatant was carefully collected and transferred to a new 1.5 mL tube. The DNA content was quantified using a NanoDrop 2000 Spectrophotometer (Thermo Scientific, Vacaville, CA, USA).

For the PCR, the 2× concentrated GoTaq^®^ Green Master Mix (Promega, Fitchburg, WI, USA) was employed, with a final volume of 15 µL. The concentrations used were as follows: 10 ng DNA and the forward and reverse primers at 0.4 µM each. The primer set (ITS1, 5′-TCCGTAGGTGAACCTGCGG-3′) and (ITS4, 5′-TCCTCCGCTTATTGATATGC-3′) was used to amplify a ~600-base pair (bp) DNA fragment of the ITS region. The amplification process commenced with an initial denaturation step at 95 °C for 5 min, followed by 35 cycles consisting of denaturation at 94 °C for 1 min, annealing at 55 °C for 2 min, extension at 72 °C for 1 min, and a final extension step of 10 min at 70 °C.

The PCR amplicons were analyzed using a 2% TBE agarose gel with SYBR Safe. The electrophoresis process was carried out at 100 V for 30 min within a Labnet Enduro Gel LX horizontal chamber (Labnet International, Inc., Edison, NJ, USA). Subsequently, the gel was imaged using a Chemi-DocTM Imaging system (BioRad, Hercules, CA, USA). The resulting fragments were then sequenced via the Sanger method, utilizing an ABI 3500xL Genetic Analyzer sequencer (Applied Biosystems, Foster City, CA, USA) with a BigDye 3.1^®^ capillary electrophoresis matrix. Finally, the obtained sequences were identified using NCBI BLAST-Nucleotide. Phylogenetic trees were constructed within the MEGA X program, employing the maximum-likelihood method with the following parameters: Kimura 2-parameter model, bootstrap analysis with 500 replicates, gamma distribution with invariant sites (G+I), a discrete number of gamma categories of 4, nearest-neighbor interchange (NNI), and a number of treatments of 16.

## 3. Results

A total of five PKC samples of the fungus growing with typical yellow-orange coloration (Figure 1) were collected and inoculated on culture medium. 

**Figure 2:**
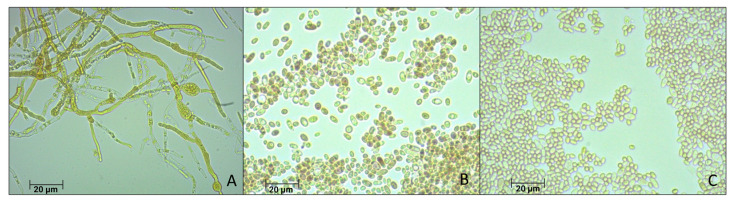
Optical microscopy at 40× magnification of the three isolated fungi from palm kernel cake of a cattle farm in Ecuador. Figure 2A–C were identified as follows: (**A**) *Geotrichum candidum* also named *Galactomyces candidus* in previous studies; (**B**) *Pichia kudriavzevii*; and (**C**) *Candida ethanolica*.

Several tens of fungi colonies were obtained and both macroscopically and microscopely, three morphological distinctive strains could be differentiated for the growth on SDA medium in the re-isolation test and optical microscopy, respectively. One strain showed a filamentous fungus. Two other unicellular yeasts strains were obtained from a mycelium-like growth on the substrate but also from the spores, both showing slightly different colors and structures (Figure 2A–C). 

**Figure 3:**
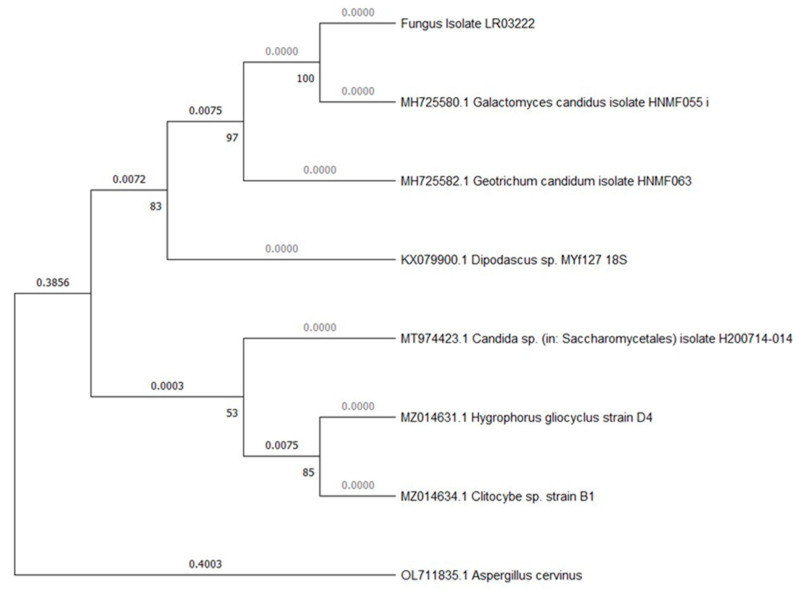
Phylogenetic tree for isolate LR03222. The phylogram shows the close relationship between our isolate and the sequences of *Geotrichum candidum* and *Galactomyces candidus* obtained from GenBank Nucleotide (available at https://www.ncbi.nlm.nih.gov/nuccore [accessed at 20 August 2023]). The tree shows the bootstrap values (numbers next to the vertex) and the distance between each sequence. The tree was constructed using the maximum-likelihood method, Kimura 2-parameter model and a bootstrap analysis with 500 replicates.

**Figure 4:**
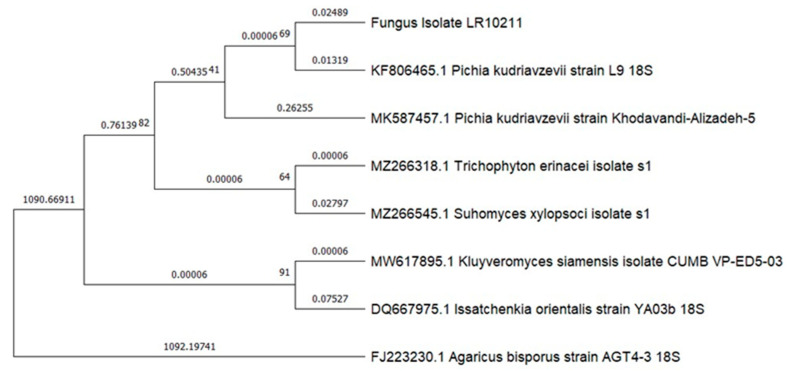
Tree for isolate LR10211. The phylogram shows the close relationship between our isolate and the sequences of *Pichia kudriavzevii* obtained from GenBank Nucleotide (available at https://www.ncbi.nlm.nih.gov/nuccore [accessed at 20 August 2023]). The tree shows the bootstrap values (numbers next to the vertex) and the distance between each sequence. The tree was constructed using the maximum-likelihood method, Kimura 2-parameter model and a bootstrap analysis with 500 replicates.

**Figure 5:**
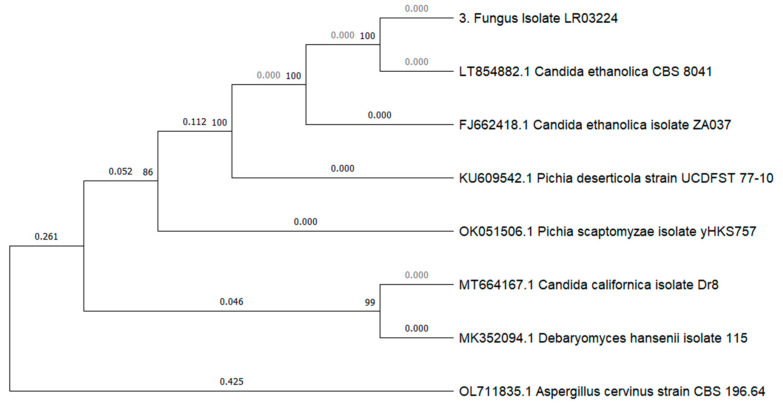
Tree for isolate LR03224. The phylogram shows the close relationship between our isolate and the sequences of *Candida ethanolica* obtained from GenBank Nucleotide (available at https://www.ncbi.nlm.nih.gov/nuccore [accessed at 20 August 2023]). The tree shows the bootstrap values (numbers next to the vertex) and the distance between each sequence. The tree was constructed using the maximum-likelihood method, Kimura 2-parameter model and a bootstrap analysis with 500 replicates.

The molecular identification, utilizing the internal transcribed spacer region (ITS sequence) and its analysis with Blastn, available at https://blast.ncbi.nlm.nih.gov/Blast.cgi, and last accessed on 16 August 2023, revealed three distinct fungal DNA sequences. The filamentous species (Figure 2A) was identified as *Geotrichum candidum* (formerly known as *Galactomyces candidus* in some older studies) with a confirmed identity value of 99.6%. The unicellular microorganisms (Figure 2B) were identified as the yeast *Pichia kudriavzevii* with a confirmed identity value of 99.6%. The growth from the orange-yellow spores was identified as the yeast *Candida ethanolica* with a confirmed identity value of 100% (Figure 2C). None of the three species are known to produce mycotoxins. The ITS sequences obtained have been uploaded to GenBank with the following accession numbers: ON478985 and ON478986 (*Geotrichum candidum* isolates), ON694346 (a *Pichia kudriavzevii* isolate), and ON478987 (*Candida ethanolica* isolate). The phylogenetic analysis conducted with the ITS sequences confirmed the identification and the relationship of our species with other nearby species and with the reference strains for *Pichia kudriavzevii* ATCC:24210 and *Candida ethanolica* ATCC:44956. (Figure 3: phylogenetic tree for *G. candidum*; Figure 4: phylogenetic tree for *P. kudriavzevii*; and Figure 5: phylogenetic tree for *C. ethanolica*).

## 4. Discussion

Palm kernel cake (PKC) is widely produced across the equatorial tropics, encompassing regions such as Southeast Asia, Africa, and South America, and stands as a vital protein and energy source for the cattle industry. However, various factors, including storage conditions, humidity levels, and high temperatures, make PKC susceptible to invasion with mycotoxin-producing fungi, posing potential threats to both cattle and human health [12,21]. 

The “yellow-orange” fungus in PKC pools alerted farmers to potential economic losses from mycotoxins and human health risks. In general, the color of fungi or the spores is not directly related to toxin production. Fungal species can vary widely in color, and while some toxigenic fungi may have characteristic colors, it is not a reliable indicator of toxin production [22,23]. The ability of a fungus to produce toxins depends on various factors such as species, environmental conditions, and genetic makeup. For example, certain fungi like *Aspergillus flavus*, which can produce aflatoxins, may have yellow-greenish spores, but not all yellow-greenish fungi produce aflatoxins. Similarly, some molds that produce mycotoxins may not display any particular coloration. Therefore, while color can sometimes provide clues about fungal species, it is not a definitive indicator of toxin production. Toxin production is best determined through laboratory testing or an analysis specific to the fungal species in question. The three species isolated in this current study, including the one producing orange-yellow spores, were determined to be unrelated to mycotoxin-producing fungi. The three species typically inhabit environments such as soil, milk, plant tissues, water, air, and the digestive tracts of mammals [24,25]. 

To our knowledge, our study marks the first of its kind in Ecuador or South America, focusing on the identification of potential mycotoxin-producing fungi in PKC used for cattle feeding. Recognizing the presence of pathogenic or mycotoxin-producing fungi is paramount due to the broad-reaching implications for animal welfare and food safety. Despite this importance, there exists a lack of surveillance and comprehensive studies regarding mycotoxin-producing fungi in Ecuador. This significant knowledge gap served as the primary motivation behind our research effort.

### 4.1. Candida ethanolica

The molecular identification of the fungus with yellow-orange spores found in the PKC storage outbuilding, *C. ethanolica*, refers to a yeast species known for its ability to ferment ethanol. *Candida* species, in general, can be opportunistic pathogens, primarily causing infections in individuals with compromised immune systems or in specific clinical settings. However, *C. ethanolica* is not among the more common opportunistic pathogens within the *Candida* genus. This yeast demonstrates a close genetic relationship with *Pichia deserticola*. Both share genetic sequences in their ITS regions, exhibiting only 5.6% differentiation between them [26]. *Pichia deserticola* has gathered attention for its potential applications in preventing and treating postharvest diseases in fruits, particularly for the control of *Penicillium, Botrytis, Alternaria*, and *Aspergillus* species [27,28]. Moreover, *C. ethanolica* has been shown to possess antifungal properties and can potentially inhibit the growth of filamentous fungi like *Aspergillus* and *Penicillium* species. These antifungal properties make *C. ethanolica* a candidate for biological control or biocontrol strategies against pathogenic fungi in various applications, such as agriculture, food preservation, or pharmaceuticals [27]. However, the extent of its inhibitory effects and the specific fungi it can target may vary based on experimental conditions and interactions between different microbial species. Regarding the striking yellow-orange color observed for this yeast growing on PKC, this coloration could arise from an intricate interplay between its metabolism and the substrate, potentially influenced by the notably high levels of phenolic compounds and essential oil content of PKC. Significantly, it is worth noting that the yeast’s coloration disappeared when cultured on SDA agar lacking these compounds.

### 4.2. Geotrichum candidum

This fungus, also known as Galactomyces candidus in older publications [29], is extremely common and has a worldwide distribution. G. candidum is a filamentous fungus characterized by branching hyphae that form a network of interconnected threads. It is commonly isolated from soil, air, water, milk, silage, plant tissues, and the digestive tract in humans and other mammals. G. candidum is widely used in the production of certain dairy products, including rind cheeses such as Camembert, Saint-Nectaire, Reblochon, and others. Additionally, this fungus has demonstrated antifungal capacity against Aspergillus spp. and has been shown to reduce the mycelial growth as well as the generation of conidia, which impacts fungal dispersion [27]. Furthermore, the fungus is an opportunistic pathogen for humans, with pulmonary involvement being the most frequently reported form of the disease. However, bronchial, oral, vaginal, cutaneous, and alimentary infections have also been noted [30].

### 4.3. Pichia kudriavzevii

This yeast, also known as *Candida krusei* in older publications, is both an opportunistic pathogen and an important industrial yeast. The yeast is widely distributed in nature [31] and is often encountered in spontaneous fermentations. The species is used to produce several traditional fermented foods, such as fermented cassava and cacao in Africa, fermented milk in Tibet and Sudan, and maize beverages in Colombia.

Additionally, this yeast species produces compounds that have fungicidal or fungistatic effects on various other fungi. *P. kudriavzevii* has been studied for its potential to be used as a biocontrol agent to prevent the growth of harmful fungi in different contexts, such as in agricultural settings or in food preservation [32]. However, the inhibitory effects of this yeast can depend on factors such as the specific strains of both this yeast and the target fungi, as well as environmental conditions. While *P. kudriavzevii* can have antagonistic effects against certain fungi, its effectiveness and the mechanisms involved can vary.

As an opportunistic pathogen, this species is the fifth most common cause of candidemia, but it is most noteworthy for its innate resistance to the antifungal agent fluconazole, in addition to its somewhat reduced susceptibility to other drugs [33].

### 4.4. Surveillance and Control of Pathogenic and Mycotoxin-Producing Fungi

Continuous surveillance in agricultural and livestock production for mycotoxin-producing fungi is necessary. The gold standard for the detection of mycotoxins is HPLC, or liquid chromatography–tandem mass spectrometry (LC-MS/MS). The most crucial steps before the mycotoxin analysis are the extraction method and clean-up. The choice of solvents, as well as the method of extraction, contribute significantly to the success of the extraction. Due to the difficulty involved in the extraction and purification of mycotoxins in samples such as PKC, most diagnostic laboratories will not carry out this analysis [34,35,36,37]. 

The methodology and results outlined in this study demonstrate an alternative and highly accessible approach to exploring the presence of potential mycotoxin-producing fungi. Moreover, our method offers a deeper understanding of the fungal ecology thriving on distinct substrates. This aspect holds significance as it allows for a nuanced assessment of fungal diversity in various food sources, potentially indicating the presence of mycotoxins without the necessity of chromatography analysis. Polymerase chain reaction (PCR)-based diagnosis has been applied by others as an alternative assay replacing cumbersome and time-consuming microbiological and chemical methods for the detection and identification of toxin producers in the fungal genera *Fusarium*, *Aspergillus*, and *Penicillium*. A review from 2007 covers the numerous PCR-based assays, which have been published since the first description of the use of this technology to detect aflatoxin biosynthesis genes in *A. flavus*. Several other publications have used a combination of fungal culture, identification with molecular biology techniques and high-performance liquid chromatography (HPLC) to identify fungal genera and the production of mycotoxins in agarwood, grains and kernels from various agricultural products and processed meat [19,20,37,38,39,40].

Addressing mycotoxin control in food is a need in Ecuador. Two notable studies conducted in the country shed light on mycotoxins in both food and breast milk. In one study, it was revealed that aflatoxin exposure through breast milk consumption raised significant health concerns across rural and urban regions. However, mycotoxin exposure from staple cereal consumption was deemed tolerable. In another study, alarming rates of contamination were observed in paddy rice, white wheat noodles, and oat flakes [41,42]. 

In our investigation, we found that PKC serves as a habitat primarily for fermenter species of fungi, none of which were identified as mycotoxin-producing. However, some of these species are associated with human diseases. For instance, *G. candidum*, a saprophytic yeast, commonly colonizes various human regions such as the skin, respiratory tract, and gastrointestinal tract. It has the potential to induce local or disseminated diseases, known as geotrichosis, particularly affecting immunocompromised individuals. Similarly, *P. kudriavzevii*, an emerging fungal nosocomial pathogen, predominantly affects immunocompromised patients and those with hematological malignancies. Notably, it exhibits inherent resistance to fluconazole, a standard antifungal medication [30,33].

### 4.5. Measures against Fungal Invasion of PKC

The presence of mycotoxins and pathogenic fungi often stems from inadequate storage conditions in food products. Factors such as high humidity, tropical climates, and insufficient infrastructure create conducive environments for mycotoxin-producing fungi to proliferate [8]. To mitigate fungal growth on PKC, a cattle feed, it is imperative to store it in dry, cool, and well-ventilated conditions while regularly monitoring for signs of fungal development. Maintaining the moisture content of the cake below levels conducive to fungal growth is essential. Exploring natural or chemical fungicides to impede fungal growth is an option, although biological control methods are preferred due to their minimal environmental impact and reduced reliance on pesticides. A notable finding in this research is the identification of three fungi thriving on PKC that exhibit antifungal properties. [43,44]. Further investigation could explore the potential introduction of Candida species into the substrate before storage as a strategy to counter the proliferation of mycotoxin-producing fungi [43,45]. Concerning the laws and rules made by authorities to fight the spread of mycotoxins in animal feed, we suggest conducting regular studies that determine the levels of mycotoxins and the presence of mycotoxin-producing fungi in animal food. It is crucial to put these measures into action quickly to reduce the risk of mycotoxins contaminating human food through animal feed.

## 5. Conclusions

The primary objective of this study was to utilize microbiological and molecular techniques to identify the orange-yellow fungus growing in the storage areas of palm kernel cake. Through this effort, the research established a robust method for detecting and monitoring fungi that may produce harmful substances known as mycotoxins. This is especially vital in tropical settings like Ecuador where palm oil is cultivated and where the access to analytics techniques such as HPLC is poor. 

Our findings revealed that this alarming-colored fungus does not produce mycotoxins. Furthermore, we demonstrated that we can cultivate, identify, and exclude mycelium growth or spores as mycotoxin-producing species using simple microbiology and molecular methods. All three species that we isolated possess antifungal properties, rendering them potential candidates for biological control strategies against pathogenic fungi [27], and of course considering the potential pathogenic risks for humans associated with some of them. 

PCR-based detection of mycotoxin-producing fungi was considered a valid method. Numerous diagnostic PCR-based technologies, as reviewed in reference [46], are now available for detection in complex food and feed matrices. Compared to other conventional methods of fungal identification, PCR-based detection technologies offer greater accuracy, sensitivity, rapidity, cost-effectiveness, safety, and portability for detecting toxigenic fungi in food and feed matrices. In the future, we can develop a technique as described in reference [19], where a multiplex polymerase chain reaction (PCR) strategy was established for rapid identification of mycotoxigenic fungi by detecting fungal species containing species-specific and mycotoxin metabolic pathway genes.

Additionally, we recommend cattle farmers to take measures to prevent the proliferation of microorganisms in cattle feed. This concern is particularly significant due to the potential transmission of mycotoxins to humans through the food chain if cattle become infected. Storage in dry, cool, and well-ventilated conditions and regularly checking for signs of fungal growth are vital [47].

## 6. Limitations of This Study

This study has some limitations. It is possible that certain fungi were not successfully cultivated on SDA, potentially leading to the omission of pathogenic or mycotoxin-producing fungi. To gain a more comprehensive understanding of the fungal presence in PKC, employing various selective fungal culture media such as malt extract, brain heart infusion medium, and chromogenic agar could provide a more precise enumeration of fungi. Additionally, this approach could aid in distinguishing colonies that share similar appearances. 

We acknowledge that our study may not have detected all fungi present on the substrate, and it is possible that some mycotoxin-producing fungi were not directly observable. However, our findings provide assurance to farmers that this specific orange-yellow fungus does not produce mycotoxins, thus offering valuable insights for agricultural practices and food safety.

For the direct detection of multiple fungus species with PCR, we tried to conduct direct PCR using ITS primers on a DNA sample extracted from PKC. Unfortunately, gel analysis did not yield distinctly separated bands, and subsequent sequencing of the product revealed overlapping sequences from various species. To address the challenge of detecting mycotoxin-producing genera in PKC, one potential solution could be the development of a multiplex PCR approach. This method, akin to the one we devised and utilized for *Prototheca* species [48], involves tailored primers designed for the four genera known to produce mycotoxins. Implementing such a technique holds promise for facilitating the direct detection and identification of fungal growth in PKC in future investigations.

Moreover, another restriction of this research relates to the reliance on endpoint PCR and Sanger sequencing of a fragment of the ITS region from a limited number of cultivated fungi or their spores for species identification. This methodology does not permit the identification of the entire fungal community [49,50]. Next-generation sequencing (NGS) offers novel methods for detecting fungal communities, surpassing microbial culture-based methods. However, due to its associated costs, NGS remains beyond the reach of most laboratories. In light of this challenge, we encourage the enhancement traditional techniques, such as those employed in this study, to provide a cost-effective alternative for identifying primary mycotoxin-producing fungi.

## Data Availability

Data are contained within the article.
